# Explainable artificial intelligence for the automated assessment of the retinal vascular tortuosity

**DOI:** 10.1007/s11517-023-02978-w

**Published:** 2023-12-07

**Authors:** Álvaro S. Hervella, Lucía Ramos, José Rouco, Jorge Novo, Marcos Ortega

**Affiliations:** 1 Centro de Investigación CITIC, Universidade da Coruña, A Coruña, Spain; 2 Grupo VARPA, Instituto de Investigación Biomédica de A Coruña (INIBIC), Universidade da Coruña, A Coruña, Spain

**Keywords:** Blood vessels, Eye fundus, Ophthalmology, Deep learning, Genetic algorithms

## Abstract

**Abstract:**

Retinal vascular tortuosity is an excessive bending and twisting of the blood vessels in the retina that is associated with numerous health conditions. We propose a novel methodology for the automated assessment of the retinal vascular tortuosity from color fundus images. Our methodology takes into consideration several anatomical factors to weigh the importance of each individual blood vessel. First, we use deep neural networks to produce a robust extraction of the different anatomical structures. Then, the weighting coefficients that are required for the integration of the different anatomical factors are adjusted using evolutionary computation. Finally, the proposed methodology also provides visual representations that explain the contribution of each individual blood vessel to the predicted tortuosity, hence allowing us to understand the decisions of the model. We validate our proposal in a dataset of color fundus images providing a consensus ground truth as well as the annotations of five clinical experts. Our proposal outperforms previous automated methods and offers a performance that is comparable to that of the clinical experts. Therefore, our methodology demonstrates to be a viable alternative for the assessment of the retinal vascular tortuosity. This could facilitate the use of this biomarker in clinical practice and medical research.

**Graphical abstract:**

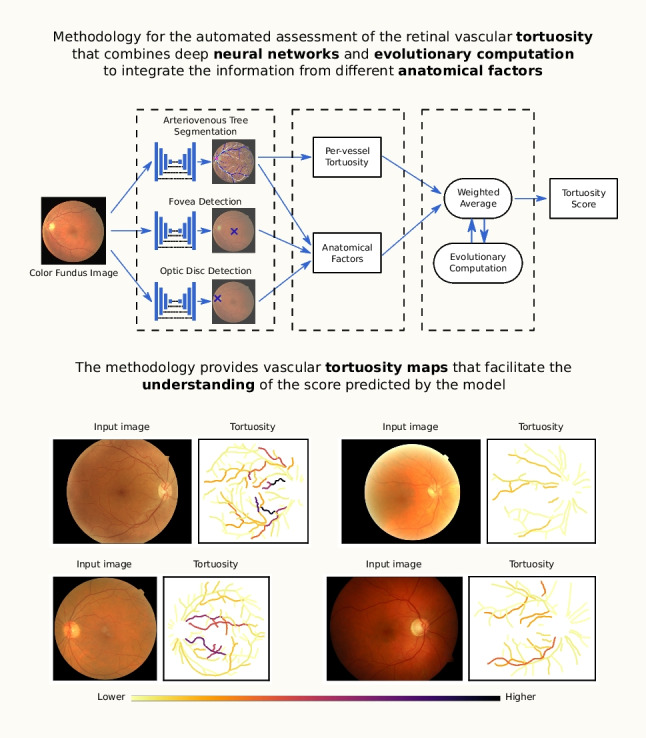

## Introduction

**Fig. 1:**
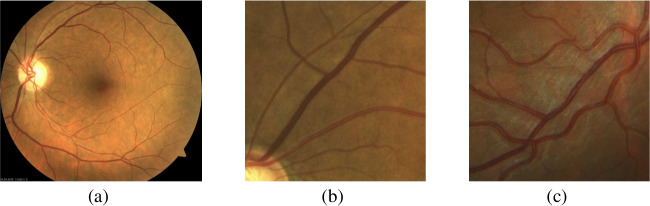
Representative examples of (a) color fundus image, (b) non-tortuous blood vessels, and (c) tortuous blood vessels

The retinal vascular tree is a complex network of arteries and veins that spread out throughout the retina [4, 35]. The analysis of this vascular network is valuable for the study and diagnosis of numerous health conditions, including both ophthalmic and systemic diseases [4, 35]. Additionally, in contrast to other parts of the human body, the eye allows the study of the vascular system in vivo and without invasive procedures [10, 35]. In that regard, color photographs of the eye fundus, such as the one depicted in Fig. 1, can be obtained using specialized fundus cameras. This is an affordable equipment that is commonly available in ophthalmic services worldwide. In that sense, color fundus images are considered a reference standard for the analysis of the retina and, consequently, numerous efforts have been dedicated to the study of the vascular network in these images [35, 47].

Different characteristics and abnormalities of the retinal vasculature have been studied as potential clinical biomarkers [35, 47]. Among these, the vascular tortuosity stands out as one of the most prominent. The retinal vascular tortuosity is an excessive bending and twisting of the blood vessels in the retina. Figure 1 depicts representative examples of tortuous and non-tortuous blood vessels. This abnormal curvature along the course of the blood vessels has been identified as a relevant biomarker for numerous health conditions, such as e.g. diabetic retinopathy [9, 42], cardiovascular disease [30, 46], or Alzheimer’s disease [2, 3]. Therefore, the clinical assessment of the retinal vascular tortuosity in color fundus images presents a great potential for diagnostic purposes.

Despite its potential applications, the use of the retinal vascular tortuosity as biomarker in the clinical practice is hampered by the difficulty of producing an objective and reliable assessment [37, 39]. The manual assessment of the vascular tortuosity from color fundus images is a tedious and time-consuming process. The complete assessment requires not only to identify potential tortuous blood vessels but also to assess the importance of each of these vessels in the global context of whole eye [16, 24, 25]. In that regard, depending on the affected blood vessels, a low level of tortuosity could be considered non-referable by the clinicians [37, 39]. Additionally, a low degree of twisting and bending in some vessels could also be considered within normality. Therefore, the manual assessment of the vascular tortuosity from color fundus images is strongly conditioned by the clinicians’ experience in the identification and study of these kinds of vascular abnormalities. These challenges motivate the development of image analysis algorithms for the automated assessment of the retinal vascular tortuosity, aiming to produce a reliable and time-efficient analysis.

Over the recent years, several computational approaches have been proposed for the automatic assessment of the retinal vascular tortuosity [33, 38, 39, 44]. Many of these works have been focused on the development of quantitative metrics for the measurement of the tortuosity of individual blood vessels. These metrics are usually based on the mathematical representation of the vessel course. In that regard, the tortuosity of an individual vessel segment is usually defined in terms of the geometrical characteristics of the vessel’s centerline, such as e.g. the curvature [33, 44], the inflection points [15, 33], or the arc and chord lengths [15, 17]. Following these approaches, the global tortuosity score of the whole image can be obtained by aggregating the individual tortuosity values of each vessel segment. This aggregation is usually performed by means of a weighted average, using the arc lengths of the vessel segments as weighting factors [15, 39]. In this case, the importance of each vessel in the global context of the whole eye is given solely by its length. However, experienced clinicians usually take into account additional anatomical characteristics of the eye, performing a more complex reasoning to assess the importance of each individual vessel [16, 24, 25]. In that line, Ramos et al. [38] propose the use of additional anatomical factors to weight the importance of each individual vessel, such as the caliber of the vessel, whether the vessel is an artery or vein, and the distances to representative anatomical structures, as the optic disc and the fovea. The inclusion of this additional domain-related knowledge in the algorithm demonstrates to provide a better prognostic performance, obtaining estimations that are better aligned with the criteria of the clinicians.

Previous methods have demonstrated to be able to provide adequate results for the automated assessment of the retinal vascular tortuosity [38]. However, these methods are limited by the use of classical image processing techniques for the extraction of the blood vessels and other relevant anatomical structures from the images. In that regard, the detection and analysis of the different anatomical structures in color fundus images is particularly challenging due to the photographic nature of these images as well as the varied characteristics of the retinal anatomy [4]. Firstly, the appearance of color fundus images can be affected by changes in the capture device, the illumination, or the expertise of the operator. Secondly, the appearance of the anatomical structures can be affected by the presence of several diseases. In that regard, the vascular tortuosity itself involves a substantial change in the morphology of the blood vessels, which can affect the performance of the algorithms. Previous works have addressed the extraction of retinal blood vessels using classical methods, such as the analysis of the level-set extrinsic curvature [1, 34], which allows to directly obtain the centerlines of the vessels. However, in comparison with modern Convolutional Neural Networks (CNN), these methods usually offer a lower performance and are less robust to changes in the acquisition of the images or the presence of pathologies. Therefore, some relevant tortuous vessels may be left undetected, compromising the assessment of the vascular tortuosity for the whole eye.

Additionally, in [38], classical hand-engineered techniques are also used for the extraction of other anatomical characteristics, which are required to compute the different anatomical factors. For instance, the detection of the optic disc is based on the premise that this region is usually the brightest in the image. Additionally, edge detection filters and a Hough transform are used to detect the circular shape of this structure. The optic disc location is used as reference point in [38] for both the detection of the fovea, using correlation filters, and the measurements of relevant properties of the previously extracted blood vessels. Thus, an error in the detection of the optic disc would also impact these subsequent analyses. In that regard, besides the extraction of the blood vessels, particularly their centerlines, the methodology of Ramos et al. [38] requires an additional process for the measurement of the caliber and the classification of the vessels as either arteries or veins. These two tasks are performed at several discrete points for each extracted blood vessel, performing a local segmentation around each point using active contour models and analyzing the intensity profiles across the vessel. Similarly to the extraction of the blood vessels, although these methods can provide adequate results under controlled conditions, modern CNNs demonstrate to be more effective and reliable alternatives [31, 32].

Deep learning algorithms have shown their effectiveness and versatility solving numerous computer vision problems. In particular, in the field of retinal image analysis, DNNs usually represent the state-of-the-art for the segmentation and detection of relevant structures in the images as well as for the diagnosis of relevant health conditions [28]. However, although DNNs have shown to be able to achieve remarkable results in the diagnosis of numerous diseases, their application in clinical practice is still limited due to the lack of understanding of their predictions [14, 29]. In that regard, there is an increasing interest in the development of interpretable automated methods for the diagnosis as well as automated methods for the estimation of clinical biomarkers [14, 27]. These biomarkers, such as the retinal vascular tortuosity, could be used as aid for the clinicians or be integrated in more complex computer-aided diagnosis systems.

In this work, we propose a robust and explainable methodology for the automated assessment of the retinal vascular tortuosity. In order to provide a reliable assessment of this biomarker, we follow a comprehensive formulation of the global tortuosity score taking into account several anatomical factors. For that purpose, the proposed methodology first performs the robust extraction of the complete arteriovenous tree, the optic disc, and the fovea using specialized neural networks. The extracted anatomical structures are used for the computation of the tortuosity value as well as the relevant anatomical factors of each individual blood vessel. Then, the global tortuosity score is obtained by integrating the information from all the blood vessels in the image. The weighting coefficients required for this integration are automatically adjusted using evolutionary computation. Besides the estimation of the global tortuosity score, the proposed methodology also provides visual representations of the tortuosity values as well as the contribution of each individual blood vessel to the final score. These visual representations directly explain the predicted tortuosity score, hence allowing to understand the decision of the model. In order to validate the proposed methodology, we conduct several comparative experiments including previous methods and we also compare the performance of our approach against the criteria of different clinical experts. These experiments are conducted on a dataset of color fundus images from diabetic patients. This dataset was explicitly gathered for the purpose of evaluating the automated assessment of the retinal vascular tortuosity. In order to ensure a high-quality gold standard, the dataset was annotated by a group of 5 clinical experts, including a joint annotation session to elaborate the final consensus ground truth.

Finally, it is worth noting that our proposal represents the first approach that integrates deep learning techniques into the computation of the global tortuosity score. The motivation for this is to provide a more robust extraction of the relevant anatomical structures. The robust extraction of these structures under challenging conditions is key for a reliable and trustworthy assessment of the retinal vascular tortuosity. Moreover, the use of evolutionary computation techniques for the adjustment of the required coefficients in the calculus of the tortuosity score further improves the robustness and effectiveness of the developed model. Additionally, our proposal offers these advantages while also providing complete visual explainability of the predictions. This is a valuable characteristic for the potential adoption of the proposed methodology in clinical practice and medical research, where the understanding of the predictions made by the models is a critical factor.

**Fig. 2:**
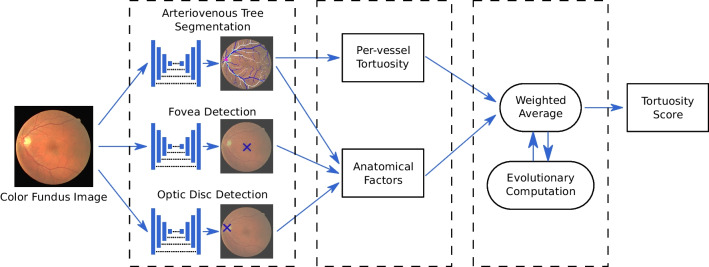
Diagram depicting the proposed methodology for the automated assessment of the retinal vascular tortuosity

**Fig. 3:**
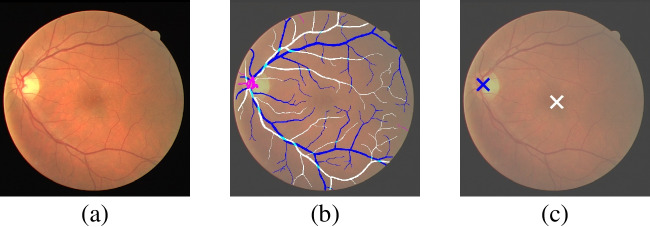
Example of color fundus image showcasing the anatomical structures of interest. (a) Original color fundus image. (b) Blood vessels maps overlaid over the original image. White denotes arteries whereas dark blue denotes veins. Meanwhile, light blue denotes overlapping between arteries and veins and pink denotes uncertain regions. (c) Locations of the optic disc and the fovea overlaid over the original image. Dark blue denotes the optic disc location whereas white denotes the fovea location

## Methods

The proposed methodology for the automated assessment of the retinal vascular tortuosity is summarized in the diagram of Fig. 2. This methodology applies the tortuosity formulation proposed in [38], which makes use of different anatomical factors to weight the importance of each individual blood vessel towards the global tortuosity score. This is intended to better emulate the process followed by experienced ophthalmologists in clinical practice. The proposed methodology is divided into three different parts. First, anatomical structures that are relevant for the computation of the tortuosity score are extracted from the image. In order to perform a reliable extraction, we use different neural networks that are specialized in each individual anatomical structure. Then, the information of these structures is used to compute the tortuosity value for each individual vessel as well as the anatomical factors that are relevant to assess the importance of each vessel within the global tortuosity. Finally, the global tortuosity score for each image is computed by aggregating the per-vessel tortuosity values. This is performed by weighting the importance of each vessel in function of the different anatomical factors. The optimization of the involved weighting parameters is performed using evolutionary computation.

### Extraction of anatomical structures with deep neural networks

The computation of the global tortuosity score requires the previous computation of individual vessel segments as well as several anatomical factors for each individual vessel. These anatomical factors are: the caliber of the vessel, whether the vessel is an artery or vein, the distance to the optic disc, and the distance to the fovea. In order to address these requirements, we perform the extraction of the following anatomical structures from the images: the arteriovenous tree, the optic disc, and the fovea. Additionally, in comparison to previous works, we perform the segmentation and classification of the complete arteriovenous tree, which allows for a better estimation of the tortuosity values and the required anatomical factors. Figure 3 depicts a representative example of color fundus image showcasing these different structures.

The arteriovenous tree consists of the blood vessels (arteries and veins) that supply and transport blood throughout the retina. Therefore, this structure can be divided into both an arterial tree and a venous tree. The extraction of the arteriovenous tree is crucial for the assessment of the tortuosity because it provides the vessel segments for which the tortuosity values are originally computed. Additionally, it also provides relevant information such as the caliber of the vessels or whether any vessel is an artery or a vein. In contrast, the optic disc represents the connection point between the optic nerve and the retina. In this region, the blood vessels naturally curve to enter and leave the retina, hence the vessel segments in the optic disc must be discarded from the analysis of the tortuosity. Additionally, the distance between each individual vessel and the optic disc is also relevant for the assessment of the tortuosity. Finally, the fovea is a small spot in the center of the posterior portion of the retina that is responsible for the sharp central vision. In this case, the distance between each individual vessel and the fovea is also relevant for the assessment of the tortuosity. We address the extraction of these anatomical structures using deep learning techniques, which are expected to provide better performance and reliability than previous alternatives, especially in the more challenging scenarios. In particular, we use DNNs that are specialized for each individual structure and are trained following state-of-the-art approaches. The particular approach followed for each anatomical structure is described below.

#### Arteriovenous tree

In this work, the extraction of the arteriovenous tree follows the approach proposed in [32]. In particular, we formulate the problem as a multi-label segmentation and train a DNN to predict three independent output maps: arteries, veins, and blood vessels. The multi-label formulation means that the output maps of the network are not mutually exclusively, i.e. the same pixel can be predicted to belong to more than one class (e.g. artery and vein at the same time). This is relevant to the problem at hand because arteries and veins intersect throughout the image, resulting in crossing points where only one of them is visible. If the network were to predict only the visible class (artery or vein), the resulting maps would be inadequately broken apart in several points (the crossings). In contrast, the multi-label approach has the potential to provide full artery and vein maps.

The network training is performed using binary cross-entropy as loss function for each individual output map. The global training loss is obtained as the sum of the three individual per-map losses. In particular, the global training loss can be defined as:

1 $$\begin{aligned} \mathcal {L}_{AVtree} = \sum _c^C \sum _{\Omega _c} - \ y_{c} \ log(p_{c}) - (1-y_{c}) \ log(1-p_{c}) \end{aligned}$$

where $C=\{ Arteries, Veins, Vessels \}$ denotes the set of the three considered classes, $\Omega _c$ the set of pixels within the Region Of Interest (ROI) of class *c*, $p_c$ the predicted value for class *c*, and $y_c$ the ground truth value for class *c*. The predicted values $p_c$ are bounded in the range [0, 1] by using a sigmoid function in the final layer of the network. The particular ROI for each class will be given by the annotations of the training dataset [32]. The existence of the different ROIs is due to the fact that, for a few vessels, it may be uncertain for the expert annotators whether the vessel is an artery or a vein. These cases will be included in the ROI of the blood vessels but will be outside of the ROI of arteries and veins (i.e. they are not considered in the arteries and veins components of the training loss)

The training of the network is performed on the publicly available RITE dataset [23]. This dataset was built upon the DRIVE dataset [43] for vessel segmentation, adding additional ground truth labels for the distinction between arteries and veins. The dataset is split into 20 training images and 20 test images. We use the 20 images of the training set. The size of the images is $768\times 584$ pixels.

Following the approach in [32], we use U-Net [41] as network architecture. This network and its variants are commonly used for biomedical image segmentation, including the extraction of the retinal vasculature [32, 40]. The network parameters are initialized following the method of He et al. [18]. Similarly to other works addressing the distinction between arteries and veins, the input images are pre-processed by performing global contrast enhancement and local intensity normalization [13, 32]. The training is performed using the Adam optimization algorithm [26] with learning rate $\alpha =0.0001$ and decay rates $\beta _1=0.9$ and $\beta _2=0.999$. The training is performed at a constant learning rate, being stopped when the validation loss does not improve for 200 epochs. As validation data, we use $25\%$ of the training set. Data augmentation is applied in the form of intensity/color transformations, affine transformations, and horizontal/vertical flipping.

The performance of the final trained model was evaluated on the test set of the RITE dataset, a standard benchmark for retinal vasculature segmentation. The model achieves 98.33% AUC in the Receiver Operator Characteristic (ROC) curve for the segmentation of the blood vessels, as well as 97.38% AUC and 98.00% AUC for the individual segmentation of arteries and veins, respectively. Additionally, after thresholding to get the class with the highest probability, the model achieves 79.12% Sensitivity and 98.65% Specificity for the segmentation of the blood vessels. Similarly, the model achieves 87.47% Sensitivity and 90.89% Specificity for the distinction between arteries and veins (considering artery the positive class).

#### Optic disc and fovea

In this work, the localization of the optic disc and the fovea follows the approach proposed in [20]. In that regard, we formulate the problem as a heatmap regression in which the target value for each individual pixel depends on its distance to the target structure. In particular, the maximum value in the heatmap corresponds to the location of the target structure and the values of the surrounding pixels progressively decrease as they are placed farther from that target. This strategy has demonstrated to be successful in the localization of different anatomical structures [20, 22]. In this work, we train DNNs to independently predict the heatmaps of the optic disc and the fovea. Then, the particular locations of these two structures can be extracted from the predicted heatmaps by finding their maximum values.

**Fig. 4:**
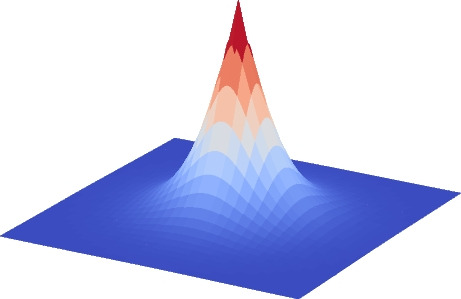
Shape of the obtained ground truth heatmaps using the hyperbolic tangent function

The network training is performed using mean squared error as loss function. In particular, the training loss can be defined as:

2 $$\begin{aligned} \mathcal {L}_{OD/Fovea} = \frac{1}{N} \sum _n^N (y_n - p_n)^2 \end{aligned}$$

where *N* denotes the number of pixel in the heatmap, $p_n$ the predicted value for pixel *n*, and $y_n$ the ground truth value for pixel *n*. As in [20], the ground truth heatmap $\textbf{y}$ presents an exponential decay with respect to the distance to the target structure. In particular, the values of the ground truth heatmap are obtained as:

3 $$\begin{aligned} y_n = 1 + tanh ( -d_n \frac{\pi }{\beta }) \end{aligned}$$

where *tanh* denotes the hyperbolic tangent function, $d_n$ the distance from pixel *n* to the target location, and $\beta$ the distance at which the heatmap values approximate to zero. The shape of these ground truth heatmaps is depicted in the example of Fig. 4.

Following the same methodology, the network is independently trained for the localization of the optic disc and the fovea. The training for the optic disc localization is performed with the images of the DRIVE dataset [43], which is described in Section 2.1.1. However, the ground truth coordinates of the optic disc locations were manually annotated for us by a clinical expert. We use the 20 images of the training set. In contrast, the training for the fovea localization is performed on the public IDRiD dataset [36], which contains the ground truth coordinates of the fovea location. The dataset is split into 413 training images and 103 test images. We use the 413 images of the training set. The original size of the images is $4288\times 2848$ pixels. However, the images were resized to a size of $858\times 570$ pixels, which was demonstrated to be sufficient for the successful localization of the fovea [20].

Following the approach in [20], we use U-Net [41] as network architecture. This network was demonstrated to be adequate for the prediction of heatmaps in several works [20, 22]. In this case, in order to facilitate the recognition of the global context in the images, the network is initialized with a pre-trained model using the self-supervised multimodal reconstruction approach proposed in [19, 21]. The training is performed using the Adam optimization algorithm [26] with an initial learning rate $\alpha =1e-5$ and decay rates $\beta _1=0.9$ and $\beta _2=0.999$. The learning rate is reduced by a factor of 10 when the validation loss does not improve for 2500 iterations. After reaching a final learning rate $\alpha =1e-7$, the training is stopped. As validation data, we use $25\%$ of the training set. Data augmentation is applied in the form of intensity/color transformations, affine transformations, and horizontal/vertical flipping.

### Computation of vessels segments, anatomical factors, and tortuosity values

The extracted anatomical structures are automatically processed to obtain the individual vessel segments and their corresponding anatomical factors. The obtained vessel segments are directly used to compute the per-vessel tortuosity values, whereas the anatomical factors will be used later for the computation of the global tortuosity score.

#### Vessel segments

The computation of the individual vessel segments starts with the binary maps of the arterial and venous trees that are predicted by the neural network. The process is the same for both types of vascular maps. First, the skeleton of the vascular tree is computed using the method of Zhang et al. [48]. This algorithm iteratively removes the border pixels of the binary vascular map until no more pixels can be removed without breaking the connectivity of the blood vessels. The resulting skeleton is a 1-pixel wide representation of the vascular tree. Then, the vessels in the optic disc region, which should not be considered for the analysis of the tortuosity, are removed from the skeleton. In particular, we remove the vessels that are within a distance *r* to the predicted optic disc location, where the value *r* depends on the size and field of view of the images. Taking into consideration the size of the images in the evaluation dataset (described in Section 2.4), we use a fixed value of $r=60$ pixels. The next step is the decomposition of the skeleton into individual vessel segments. For this step, junctions in the skeleton are detected using morphological operators. In particular, we apply a hit-or-miss transform considering all the possible rotations of the T-shaped and Y-shaped junction patterns. The detected junctions are used to split the skeleton into individual vessel segments. In general, at each junction, the skeleton is split into three different segments. However, as in [38, 39], in those cases where two potential new segments present matching directions and calibers, those two segments are kept joined together as a single vessel segment. The direction of the segments is given by their tangent lines. Meanwhile, the caliber, which is one of the anatomical factors considered for the analysis of the tortuosity, is computed as described in Section 2.2.2. Finally, each vessel segment is defined by the list of its constituent pixels, and the length of the segment is given by the number of these pixels.

#### Anatomical factors

##### Caliber

The caliber of the vessel segments is obtained by computing the Euclidean distance transform of the binary vascular maps that are predicted by the network. This operation assigns to each pixel its distance to the border of the binary vascular region. Thus, the value assigned to each pixel of the skeleton will be approximately half the caliber of the vessel at that particular section. Then, the caliber for each vessel segment is computed as the average caliber of all the sections (or pixels) in that vessel.

##### Artery-vein distinction

The probabilities of being artery or vein are obtained from the maps predicted by the network before binarization. The precise probabilities assigned to each vessel segment are computed as the average along the segment. The vessel segments are considered to belong to the class with higher probability.

##### Distance to the optic disc

The distance from any vessel segment to the optic disc is computed as the average distance from the pixels of that segment to the optic disc location.

##### Distance to the fovea

The distance from any vessel segment to the fovea is computed as the average distance from the pixels of that segment to the fovea location.

**Fig. 5:**
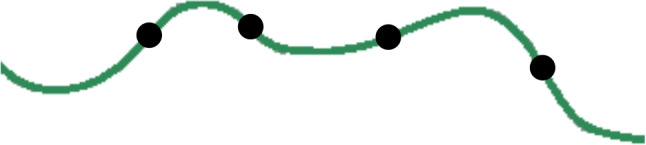
Example of tortuous vessel segment consisting of four inflection points and six subsegments. Black dots denote the inflection points

#### Tortuosity values

The tortuosity value of each individual vessel segment is computed using the metric proposed by Grisan et al. [15]. This metric has demonstrated to provide a better matching with the criteria of the ophthalmologists in comparison to other alternatives in the literature [39]. According to [15], the tortuosity of a vessel segment depends on how many times the vessel changes its convexity (or curvature sign) and how large is the amplitude of the curve that is described between every two consecutive convexity changes. In order to measure these parameters, first the vessel is smoothed using a low-pass filter that removes undesirable noise due to the discrete nature of the pixel representation [11, 17]. In particular, as in [38, 39], we apply a Savitzky-Golay filter with polynomial order 2 combined with a Gaussian filter with sigma value of 3 pixels. Then, the vessel is split at the inflection points where its convexity changes. This results in *n*-subsegments of constant-sign curvature. An example of inflection points and subsegments can be seen in Fig. 5. The amplitude of the curve in each subsegment is measured using the ratio between the arc length and the chord length of that subsegment. Finally, this information is integrated into the formal definition of tortuosity:

4 $$\begin{aligned} \tau _{v}=\frac{n-1}{L_c}\sum _{i=1}^{n}\left[ \frac{L_{csi}}{L_{xsi}}-1\right] \end{aligned}$$

where $L_c$ denotes the arc length of the whole vessel segment, $L_{csi}$ the arc length of subsegment *i*, and $L_{xsi}$ the chord length of subsegment *i*.

### Computation of the global tortuosity score using evolutionary computation

#### Formulation of the global tortuosity score

The global tortuosity score for a given image is obtained by aggregating the individual tortuosity values of the different vessel segments. In particular, taking into consideration the compositionality property of the vascular tortuosity [17], the global tortuosity score is obtained by computing the weighted mean of the per-vessel tortuosity values. Thus, the global tortuosity score is defined as:

5 $$\begin{aligned} \tau _{f} = \frac{\sum _{i=1}^{n} \tau _{vi}f_i}{\sum _{i=1}^{n}{f_i}} \end{aligned}$$

where $\tau _{vi}$ and $f_{i}$ denote the tortuosity value and the weighting factor, respectively, of the vessel segment *i*. The weighting factor $f_i$ weights the importance of each vessel segment *i* in the computation of the global tortuosity score. When no additional anatomical factors are considered, the standard approach is to use the vessel length as the weighting factor for each segment, i.e. $f_i=L_{ci}$ [38, 39]. However, it is also possible to include additional anatomical factors in the computation [38]. In that regard, we define the weighting factor $f_i$ for a given vessel segment *i* as follows:

6 $$\begin{aligned} {\begin{matrix} f_i = L_{ci} \cdot [(\omega _{AV} \cdot f_{AV/i} + (1-\omega _{AV}) \cdot (1-f_{AV/i})) \; + \\ (\omega _{Cal} \cdot f_{Cal/i}) + (\omega _{dOD} \cdot f_{dOD/i}) + (\omega _{dFov} \cdot f_{dFov/i})] \end{matrix}} \end{aligned}$$

where $\omega _{AV}$, $\omega _{Cal}$, $\omega _{dOD}$, and $\omega _{dFov}$ are the coefficients for the anatomical factors that denote the probability of being artery ($f_{AV}$), the caliber in pixels ($f_{Cal}$), the distance to optic disc in pixels ($f_{dOD}$), and the distance to fovea in pixels ($f_{dFov}$), respectively. Additionally, the term $1-\omega _{AV}$ represents the coefficient that is applied to the probability of being vein ($1-f_{AV}$). This implies that $0 \le \omega _{AV} \le 1$. As the tortuosity must be always a non-negative value, all the coefficients are subjected to the constraint $\omega _x \ge 0 \ \ \forall \omega _x \in \{\omega _{AV}, \omega _{Cal}, \omega _{dOD}, \omega _{dFov}\}$. The coefficients of the different anatomical factors are automatically selected through evolutionary computation as described below (Section 2.3.2).

#### Estimation of weighting coefficients using evolutionary computation

In order to obtain the most adequate values for the coefficients of the anatomical factors, an optimization process with evolutionary algorithms is performed. In that regard, it must be noticed that the goal of the methodology herein presented is to accurately classify the images in function of the presence of vascular tortuosity. For that purpose, a threshold $\phi _{tort}$ can be used such that retinas are considered tortuous when their global tortuosity score $\tau _f$ is superior to $\phi _{tort}$ and non-tortuous otherwise. The performance of this binary classification can be assessed by means of the Sensitivity (*Sen*) and the Specificity (*Sp*) with respect to the ground truth. In that regard, we are interested in finding the coefficients $\omega _x$ and the threshold $\phi _{tort}$ that provide both a high Sensitivity and a high Specificity. However, there is usually a trade-off between this two metrics, such that optimal solutions with higher Sensitivity usually present lower Specificity and viceversa. In this scenario, we perform a multi-objective optimization to find the solutions that are Pareto optimal with regards to Sensitivity and Specificity. This optimization process is performed with the Non-dominated Sorted Genetic Algorithm II (NSGA-II) [6], which is an genetic algorithm commonly used to solve multi-objective optimization problems [5, 12, 45].

The NSGA-II algorithm iteratively evolves the population of candidate solutions (the coefficients $\omega _x$ and the threshold $\phi _{tort}$, in this case) by performing selection, genetic crossover, and genetic mutation operations. In that regard, NSGA-II follows the general outline of a genetic algorithm using a modified survival and selection process [6]. In particular, after applying crossover and mutation operators to generate a new offspring population of candidate solutions, NSGA-II merges together the parent and offspring populations. This ensures elitism by allowing the best candidate solutions to continue to the next generation. The survival of candidate solutions in each iteration is given by their order according to two different criteria. First, the solutions are ranked according to the non-dominated front to which they belong in objective space, i.e. solutions towards the Pareto-optimal front are given precedence. Second, the solutions are sorted by their crowding distance in objective space, given precedence within the same front level to solutions in less crowded regions. This guides the selection process towards an uniformly spread-out Pareto-optimal front. Then, the selection of solutions that will be used to generate the new offspring population is performed with a binary tournament using the same criteria.

Regarding the operations to generate the new offspring populations, the crossover is performed using Simulated Binary Crossover with $\eta _c=15$ and probability $p_c=0.9$ [5, 8]. Meanwhile, the mutation is performed using Polynomical Mutation with $\eta _m=20$ and probability $p_m=0.9$ [5, 7]. The population size is 500 and the optimization is conducted until convergence [5].

The evolutionary optimization process is performed on the same dataset that we use to validate the complete methodology, which is described in detail in Section 2.4. In that regard, it must be noticed that evaluation is performed using Monte Carlo cross-validation, i.e. the NSGA-II optimization and evaluation are always performed on different subsets of the dataset. The complete evaluation procedure is described in detail in Section 2.5.

### Dataset

**Fig. 6:**
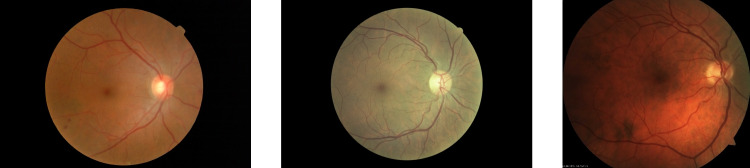
Representative examples of color fundus images in the evaluation dataset

The methodology is validated on a dataset of color fundus images that was also used in previous studies [38, 39]. The dataset consists of 200 color fundus images from a cohort of diabetic patients. The level of vascular tortuosity in these images ranges from no visible tortuosity to severe tortuosity. The images present varying sizes, ranging from 616$\times$550 to 1020$\times$680 pixels, and represent a variety of characteristics and capture conditions. Figure 6 depicts some representative examples of images in the dataset.

The annotation of the images was performed by a group of five clinical experts who are actively involved in the daily clinical practice of an ophthalmology service. The level of experience of these clinicians ranged from head of service to resident physician. The annotation process involved two individual rating rounds and a joint session where the five clinical experts discussed their discrepancies. The ratings resulting from this joint session are used as the consensus ground truth. This ground truth provides the classification of the images into two categories: non clinically-relevant and clinically-relevant vascular tortuosity. Non clinically-relevant tortuosity includes images with no tortuosity as well as images with mild tortuosity, which is a common asymptomatic anomaly in the human body [16]. Meanwhile, clinically-relevant tortuosity includes images with moderate or severe tortuosity, which is known to be associated with significant health risks [16, 25]. Given the absence of standard measurable criteria for the assessment of the tortuosity, the distinction between these categories is made by the clinicians on the basis of their accumulated knowledge and experience in the field. Table 1 provides the inter-observer agreement for the five clinical experts as well as the consensus ground truth after the final rating round.

**Table 1:** Inter-observer agreement in terms of Cohen-Kappa score for the five clinical experts

	E2	E3	E4	E5	Rc
E1	0.47	0.56	0.41	0.33	0.55
E2		0.54	0.58	0.53	0.69
E3			0.62	0.58	0.79
E4				0.58	0.65
E5					0.71

A higher value denotes greater agreement. E*n* denotes Expert number *n*, whereas Rc denotes the consensus ground after the final rating round

### Evaluation procedure

We follow the same evaluation procedure as previous works [38]. In that regard, the assessment of the global tortuosity is performed by means of a Receiver Operator Characteristic (ROC) analysis. In particular, we use the ROC convex hull curves, which depict ’Sensitivity’ against ’1 - Specificity’ for different operating points. For the experiments using evolutionary computation, the curves are constructed as in [38]. First, we measure the sensitivity and specificity in the test set of the different Pareto-optimal solutions provided by the evolutionary optimization process. In this case, each individual solution represents a different operating point in ROC space, as each solution includes a classification threshold to be applied over the obtained tortuosity score. The final curve is constructed by computing the convex hull of the different operating points. Besides depicting the curve, we also use the Area Under the Curve (AUC) as evaluation metric. The experiments are conducted using Monte-Carlo cross-validation, hence the optimization and the evaluation are always performed in independent subsets of the dataset. In particular, we perform 10 repetitions of the experiments with 10 independent random splits of the dataset, using 80% of the data as training set and 20% as test set. We plot the average curves and report the AUC values as ’mean ± standard deviation’.

We also perform some experiments without using the anatomical factors or using only one of them at each time. In general, these cases do not require the use of weighting coefficients and, therefore, no optimization process is required. Consequently, the ROC analysis is performed by directly applying different thresholds over the global tortousity score, such that each threshold value represents a different operating point in ROC space. For consistency with the evaluation using evolutionary computation, the final curve is also constructed by computing the convex hull of the different operating points. In this case, as no optimization is required, the evaluation is performed in a single step using 100% of the data as test set.

In order to provide an additional frame of references for the validation, we also evaluate the performance of each individual clinical expert when compared against the consensus ground truth. Each of the experts will be represented by a single operating point in ROC space.

**Fig. 7:**
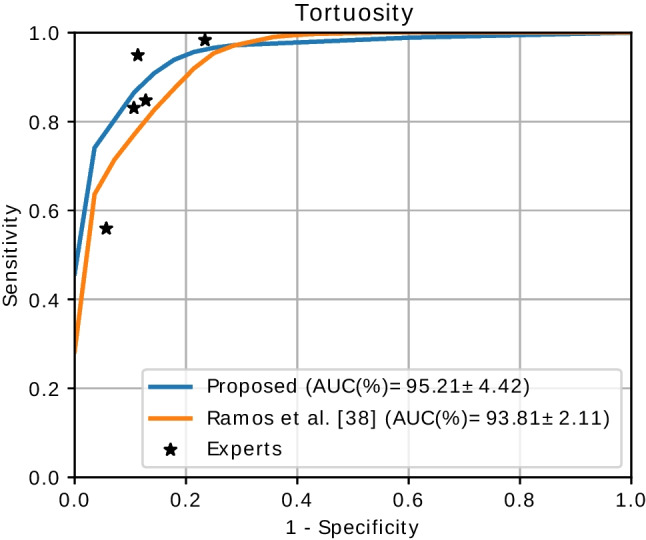
Results and comparison for the assessment of the retinal vascular tortuosity. The plots represent the average curve for each method using Monte-Carlo cross-validation

## Results and discussion

### Evaluation of the proposed methodology

In order to validate the proposed methodology, we perform a comparison against the method of Ramos et al. [38] and the different clinical experts. The method of Ramos et al. [38] follows the same formulation of the global tortuosity score that is applied in our work. In that regard, this method also requires the selection of optimal weighting coefficients and thresholds using evolutionary computation. To provide a fair comparison, we perform experiments using the same training-test splits for both methods. Figure 7 depicts the results of our experiments. These results show that the proposed methodology clearly outperforms the previous method. Firstly, our proposal achieves a greater AUC value, which denotes a better overall performance. Secondly, as it is shown in the depicted curves, the improvement is particularly notorious in the region with both high sensitivity and high specificity, which is of especial interest due to its well-balanced performance. Additionally, whereas the method of Ramos et al. [38] only outperforms one of the clinical experts, our proposal is able to surpass the performance of three individual experts. This shows that the proposed methodology could be a valuable tool for the assessment of the retinal vascular tortuosity in real clinical settings, where the combined opinion of multiple clinicians is usually not available.

The improvement of our proposal with respect to the method of Ramos et al. [38] can be explained by the use of DNNs for the extraction of the relevant anatomical structures, instead of hand-engineered classical computer vision approaches. In that regard, the reliable extraction of the arteriovenous tree, the optic disc, and the fovea is key not only to the computation of the individual vessel segments but also to the computation of the different anatomical factors.

### Evaluation without anatomical factors

In the literature, some previous works have approached the assessment of the retinal vascular tortuosity without considering the use of anatomical factors. These kinds of approaches are expected to provide a lower performance. However, avoiding the use of anatomical factors can also result in a more computationally efficient approach. Therefore, it is of interest to study whether the performance benefit due to the use of DNNs also translates to these more simple settings. To that end, we perform additional experiments to evaluate our proposal without the anatomical factors and compare the performance of this variant against equivalent previous methods. In these experiments, the global tortuosity score is computed using only the vessel length to weight the contribution of each individual vessel segment to the global tortuosity. Consequently, the optimization process to select the different weighting coefficients and thresholds is not required.

**Fig. 8:**
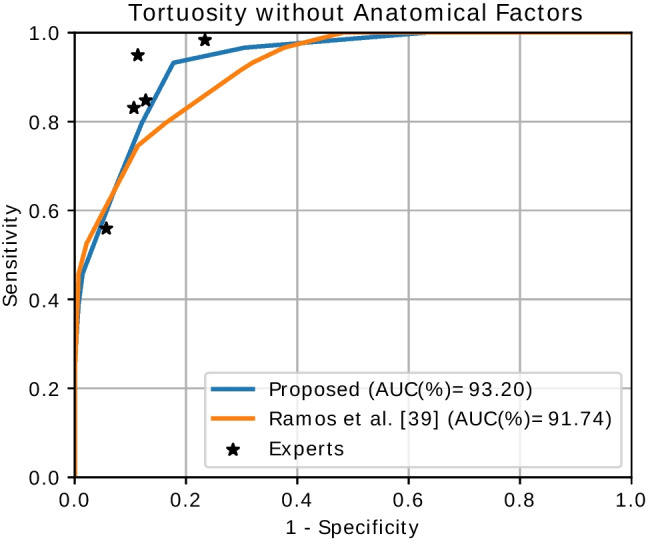
Results and comparison for the assessment of the retinal vascular tortuosity without anatomical factors

Figure 8 depicts the results of our experiments without the anatomical factors. In this case, we compare the performance of our proposal against a previous method of Ramos et al. [39] that do not requires anatomical factors either. The results show that our proposal also provides adequate results in this setting and outperforms the method of Ramos et al. [39]. However, in this case, the performance is lower than the one achieved using our complete methodology (see Fig. 7). Firstly, these results demonstrate that the extraction of the anatomical structures with DNNs also represents an advantage when no anatomical factors are considered. In this case, the DNNs were used to extract the optic disc and the vascular map (but not the arterial and venous maps). These structures are key to compute the vessel segments that are required for the calculus of the global tortuosity. Secondly, the results also demonstrate that the use of anatomical factors in our complete methodology provides additional relevant knowledge that is useful for the assessment of the vascular tortuosity. As shown in the depicted curves, taking into consideration this additional anatomical knowledge is key to achieve a performance that is highly competitive with the clinical experts.

### Analysis of the different anatomical factors in the methodology

Given that the proposed methodology makes use of a variety of anatomical factors to improve the performance, in this section we study the individual impact of each of these factors in the assessment of the vascular tortuosity. For that purpose, we perform additional experiments including the anatomical factors one at a time. In the case of the caliber, the distance to the optic disc, and the distance to the fovea, the weighting factor of each vessel is directly computed as the product of the vessel length and each of these magnitudes (caliber, distance to optic disc, and distance to fovea). Thus, the optimization process to select the different weighting coefficients and thresholds is not required. In that regard, the performance when using any of these anatomical factors is evaluated using standard ROC analysis (as in Section 3.2). In contrast, when using the artery-vein distinction, it is necessary to estimate the weighting coefficient of arteries against veins to compute the weighting factor of each vessel. This is performed using the same evolutionary optimization process as if all the anatomical factors were considered. Although, in this case, the artery-vein distinction is the only factor included in the computation. In that regard, the performance when using artery-vein distinction is evaluated using ROC analysis with Monte-Carlo cross-validation (as in Section 3.1).

**Fig. 9:**
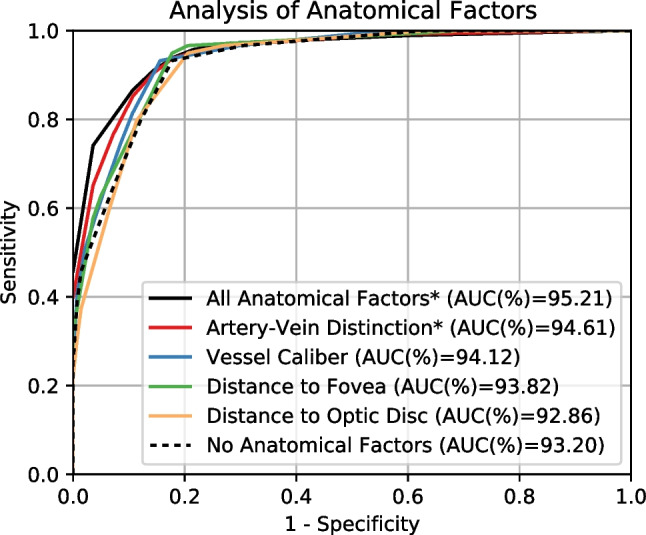
Results for the assessment of the retinal vascular tortuosity using the individual anatomical factors. * denotes that the method performs the adjustment of weighting coefficients with evolutionary computation and the evaluation is performed using Monte-Carlo cross-validation

Figure 9 depicts the results of the experiments using the anatomical factors one at a time. We also include as reference the results corresponding to the use of all and none of the anatomical factors. Additionally, Table 2 depicts the results in terms of AUC and relative improvement with respect to the baseline without anatomical factors. These results show that, with the exception of the distance to the optic disc, the anatomical factors also contribute to a better performance when considered in isolation. This means that these anatomical factors provide relevant knowledge for the assessment of the vascular tortuosity. Additionally, the obtained results also show a clear ranking of the anatomical factors in terms of improvement with respect to the baseline, being artery-vein distinction the anatomical factor that provides a greater improvement. Nevertheless, the combined use of all the anatomical factors in the proposed methodology is the alternative that provides the best results.

**Table 2:** Results for the assessment of the retinal vascular tortuosity using the individual anatomical factors

Method	AUC(%)	Difference (%)
All anatomical factors*	95.21	+2.15
Artery-Vein distinction*	94.61	+1.51
Vessel Caliber	94.12	+0.99
Distance to Fovea	93.82	+0.66
Distance to Optic Disc	92.86	−0.37
No Anatomical Factors	93.2	0.00

Difference (%) is computed as the relative improvement with respect to the method ’No Anatomical Factors’. * denotes that the method performs the adjustment of weighting coefficients with evolutionary computation and the evaluation is performed using Monte-Carlo cross-validation

**Table 3:** Results for the assessment of the retinal vascular tortuosity using different alternatives with and without the distance to the Optic Disc (OD)

Method	AUC(%)	Difference(%)
All Anatomical Factors*	95.21	+2.15
All but Distance to OD*	95.21	+2.15
Distance to OD	92.86	− 0.37
Distance to OD (Reversed)	92.73	− 0.50
No Anatomical Factors	93.2	0.00

Difference (%) is computed as the relative improvement with respect to the method ’No Anatomical Factors’. * denotes that the method performs the adjustment of weighting coefficients with evolutionary computation and the evaluation is performed using Monte-Carlo cross-validation

**Fig. 10:**
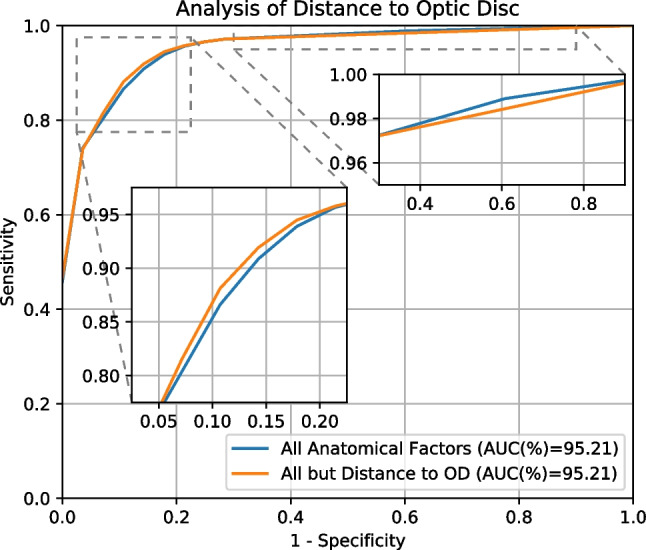
Results for the assessment of the retinal vascular tortuosity with and without the distance to the Optic Disc (OD). The evaluation is performed using Monte-Carlo cross-validation

Given the slight reduction in performance when using the distance to the optic disc alone, we perform some additional experiments to analyze whether this may have an impact in the overall performance of our proposal. In that regard, first, we evaluate our complete methodology using all the anatomical factors except the distance to the optic disc. Second, we also evaluate the performance using a reversed version of the distance to the optic disc, considering that vessels with smaller distances may need to be weighted more instead of the opposite. For these experiments, we define the reversed distance to the optic disc as $Rd_{OD} = k - d_{OD}$ where *k* is a constant term aimed to avoid negative values. In this case, we use $k=600$, which is a value slightly greater than the largest distance in the dataset. The results of these additional experiments are depicted in Table 3. Firstly, the results show that the reversed version of the distance to the optic disc also produces a lower performance than the baseline without anatomical factors. In that regard, according to our experiments, neither the distance to the optic disc nor the reversed distance correlate with the importance of the vessels for the assessment of the global vascular tortuosity. In this scenario, both the distance and the reversed distance negatively affect the performance, arguably because they introduce noise in the predictions. Secondly, the results show that our complete methodology provides a similar performance regardless of the inclusion or not of the optic disc distance as one of the anatomical factors. This indicates that the methodology is robust to the inclusion of additional anatomical factors, even when they may have a deleterious effect in the performance when considered in isolation. The explanation for this is that, during the evolutionary optimization process, the most adequate combinations of anatomical factors can be learned from the training data. In that regard, it is worth noting that the evolutionary algorithm also has the ability to suppress any of the anatomical factors by drastically reducing its weighting coefficient. However, it is also possible that the evolutionary algorithm could find some combinations of weighting coefficients for which all the anatomical factors present a positive or neutral impact, regardless of their deleterious effect when considered in isolation. In that regard, Figure 10 depicts the evaluation curves for our proposal both with and without the distance to the optic disc. These plots show that, despite the same mean AUC value, the curves are slightly different in some areas. Thus, there are some particularly cases for which the distance to the optic disc has a small positive impact. Although this is at the expense of a small negative impact in other cases. Nevertheless, given the small magnitude of these differences, the complete methodology that we propose demonstrates to be robust to the selection of the anatomical factors.

**Fig. 11:**
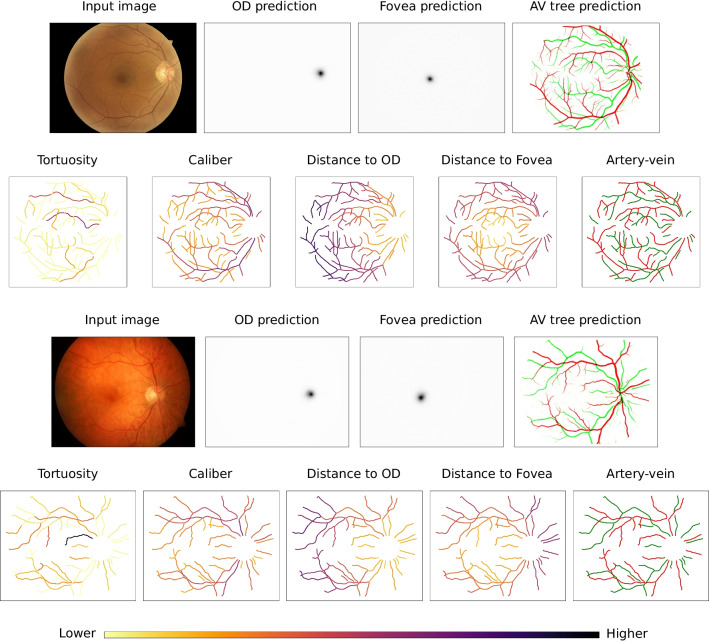
Visual representations generated using the proposed methodology for two representative examples of color fundus images. For each input image, the predictions of the neural networks are depicted in the same row. Then, for that same input image, per-vessel maps of the vascular tortuosity and the different anatomical factors are depicted in the next row. In the case of artery-vein distinction, red denotes arteries while green denotes veins. In the case of the tortuosity and the remaining anatomical factors, the colors follow the scale depited at the bottom of the Figure

### Visual examples and explainability

In order to better comprehend the proposed methodology, in this section we provide some representative visual examples. In that regard, Figure 11 depicts visualizations generated at different steps of the methodology. Firstly, for each input image, we depict the raw predictions of the DNNs, including the heatmap of the optic disc (OD), the heatmap of the fovea, and the blood vessel maps for arteries and veins. The predicted locations for the OD and the fovea are given by the position of the maximum value in the heatmaps, which should approximately correspond with the center of the depicted Gaussian blobs (the dark regions). Meanwhile, the blood vessel maps depict the individual predicted likelihoods of the arteries and veins in two different colors (red and green). In this case, dark regions denote overlapping of arteries and veins, which usually happens in the vessels crossovers. Secondly, also for each input image, we depict the computed skeleton of the retinal vasculature using a colormap to represent the values of the tortuosity and the different anatomical factors for each individual vessel segment. In particular, we provide independent colormaps for tortuosity, caliber, distance to optic disc (OD), distance to fovea, and artery-vein distinction. For the latter, the used colors match the ones of the vessel maps predicted by the network, whereas for the others the used colors follow the scale depicted in the legend of the Figure.

Regarding the predictions of the DNNs, the examples demonstrate that the trained networks are able to precisely detect the location of the optic disc and the fovea as well as extract the complete arteriovenous tree. Additionally, these satisfactory predictions are obtained for two color fundus images with very different visual characteristics, demonstrating the robustness of the networks to variations in the characteristics of the input domain. With regards to the skeleton maps depicting the per-vessel tortuosity as well as the different anatomical factors, the examples also demonstrate the successful computation of these values.

**Fig. 12:**
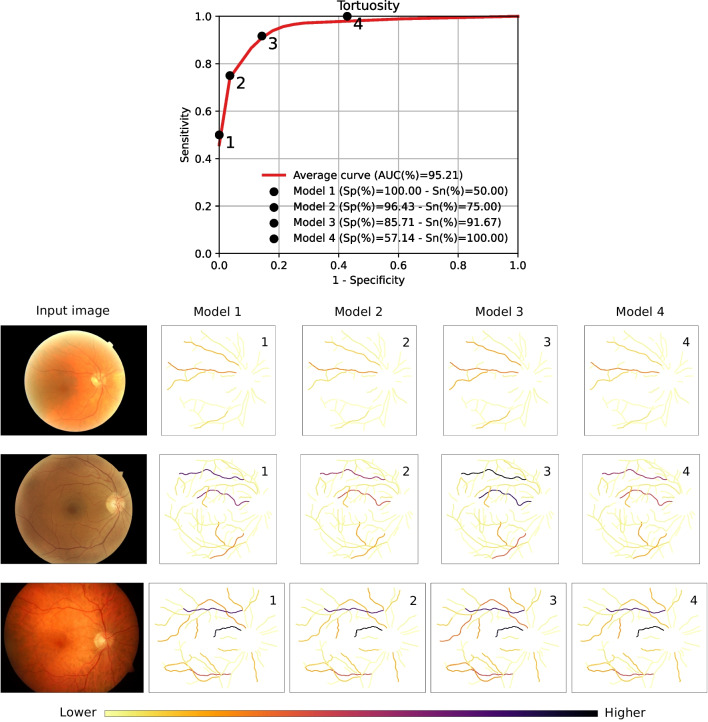
Weighted tortuosity maps that are generated using the proposed methodology for three representative color fundus images. These weighted maps represent the contribution of each individual blood vessel to the global tortuosity score. For each input image, four different maps are generated using four representative models evenly spread in ROC space. Each of the models represents a different solution from the evolutionary optimization process. The operating point of each model is described in the plot at the top of the figure, where Sp denotes Specificity and Sn denotes Sensitivity

The previous depicted visualizations can be useful for understanding the predictions of the proposed methodology. However, in order to completely explain the obtained predictions, our proposal can also provide the weighted tortuosity maps that are obtained after taking into account the different anatomical factors. It is worth noting that, depending on their anatomical characteristics, two vessels with similar tortuosity values can provide very different net contributions to the global tortuosity score. In this regard, the weighted maps represent the final contribution of each vessel to the predicted tortuosity score. Thus, they directly explain the predicted global tortuosity. Figure 12 depicts examples of these weighted maps obtained from models at different operating points in ROC space. The different models correspond to different solutions obtained from the evolutionary optimization process. In this case, we selected four representative models that are evenly spread in ROC space. The best model in terms of balanced accuracy is *Model 3*. The coefficients and the threshold that are used in this model are the following: $\omega _{AV}= 2.23 \times 10^{-1}$, $\omega _{Cal}= 8.83 \times 10^{-2}$, $\omega _{dOD}= 1.32 \times 10^{-3}$, and $\omega _{dFov}= 6.51 \times 10^{-4}$, and $\phi _{tort}= 2.46 \times 10^{-3}$. The examples show that the vessels that contribute the most are usually the same across different operating points. However, the particular contribution of each vessel differs from model to model, as each of the models is adjusted to produce an specific trade-off between specificity and sensitivity. In this regard, it is worth noting that In general, the depicted visualizations allow to explain the prediction of the global tortuosity score in terms of the individual blood vessels. This facilitates the understanding of the final predictions and, consequently, would also facilitate the adoption of the proposed methodology in clinical practice.

## Conclusions

The retinal vascular tortuosity is a relevant biomarker for systemic and ophthalmic diseases. However, the adoption of this biomarker in clinical practice is hampered by the difficulty of obtaining an objective and reliable assessment of the tortuosity. In this context, we propose a robust and explainable methodology for the automated assessment of the retinal vascular tortuosity from color fundus images. Our proposal is based on a comprehensive formulation of the retinal vascular tortuosity, which takes into account several anatomical factors to weight the importance of each individual blood vessel. In contrast to previous works, we use specialized neural networks for the extraction of the required anatomical structures. In particular, these networks focus on the segmentation and classification of the arterial and venous vascular trees, the localization of the optic disc, and the localization of the fovea. Additionally, an evolutionary optimization process is performed to obtain the most adequate weighting coefficients for each anatomical factor.

The proposed methodology is validated on a dataset of color fundus images from diabetic patients with a consensus ground truth and the annotations of five clinical experts. The obtained results show that our proposal outperforms previous automated methods and offers a performance that is comparable to that of the clinical experts. These results demonstrate that our proposal is a viable alternative for the assessment of the retinal vascular tortuosity. Moreover, besides the estimation of the global tortuosity score, the proposed methodology also allows to obtain visual representations depicting the tortuosity of each blood vessel and its contribution to the global score. This complementary information allows to easily understand the predicted global tortuosity and can potentially facilitate the adoption of our proposal in clinical practice.

Finally, the obtained results also show that our proposal is robust to the inclusion of different anatomical factors. In that regard, in this work we use the same factors that were previously deemed relevant in the literature. However, future works could explore different approaches to automatically discover the anatomical factors that are more relevant for the assessment of the retinal vascular tortuosity. Additionally, given the successful results that are achieved in this work for the assessment of the retinal vascular tortuosity, we consider that there is a remarkable potential for using this biomarker in computer-aided diagnosis pipelines and clinical practice. Thus, exploring the inclusion of this biomarker in clinical settings for the diagnosis of ophthalmic and systemic diseases is a preferential future research direction.
